# Preventing Olanzapine-Induced Weight Gain Using Betahistine: A Study in a Rat Model with Chronic Olanzapine Treatment

**DOI:** 10.1371/journal.pone.0104160

**Published:** 2014-08-01

**Authors:** Jiamei Lian, Xu-Feng Huang, Nagesh Pai, Chao Deng

**Affiliations:** 1 Antipsychotic Research Laboratory, Illawarra Health and Medical Research Institute, University of Wollongong, Wollongong, NSW, Australia; 2 Centre for Translational Neuroscience, School of Medicine, and Illawarra Health and Medical Research Institute, University of Wollongong, Wollongong, NSW, Australia; 3 Schizophrenia Research Institute, Sydney, NSW, Australia; University of Santiago de Compostela School of Medicine - CIMUS, Spain

## Abstract

Olanzapine is the one of first line antipsychotic drug for schizophrenia and other serious mental illness. However, it is associated with troublesome metabolic side-effects, particularly body weight gain and obesity. The antagonistic affinity to histamine H_1_ receptors (H_1_R) of antipsychotic drugs has been identified as one of the main contributors to weight gain/obesity side-effects. Our previous study showed that a short term (2 weeks) combination treatment of betahistine (an H_1_R agonist and H_3_R antagonist) and olanzapine (O+B) reduced (−45%) body weight gain induced by olanzapine in drug-naïve rats. A key issue is that clinical patients suffering with schizophrenia, bipolar disease and other mental disorders often face chronic, even life-time, antipsychotic treatment, in which they have often had previous antipsychotic exposure. Therefore, we investigated the effects of chronic O+B co-treatment in controlling body weight in female rats with chronic and repeated exposure of olanzapine. The results showed that co-administration of olanzapine (3 mg/kg, t.i.d.) and betahistine (9.6 mg/kg, t.i.d.) significantly reduced (−51.4%) weight gain induced by olanzapine. Co-treatment of O+B also led to a decrease in feeding efficiency, liver and fat mass. Consistently, the olanzapine-only treatment increased hypothalamic H_1_R protein levels, as well as hypothalamic pAMPKα, AMPKα and NPY protein levels, while reducing the hypothalamic POMC, and UCP_1_ and PGC-1α protein levels in brown adipose tissue (BAT). The olanzapine induced changes in hypothalamic H_1_R, pAMPKα, BAT UCP_1_ and PGC-1α could be reversed by co-treatment of O+B. These results supported further clinical trials to test the effectiveness of co-treatment of O+B for controlling weight gain/obesity side-effects in schizophrenia with chronic antipsychotic treatment.

## Introduction

Second generation antipsychotic drugs have surpassed first-generation agents as the first line of treatment for schizophrenia. Among them, olanzapine is one of the most widely prescribed antipsychotic drugs to treat schizophrenia and other serious mental disorders such as bipolar disorder, dementia, major depression, and Tourette's syndrome due to its enhanced tolerability [1]–[6]. Unfortunately, olanzapine, along with clozapine, have the highest risk for substantial weight gain, obesity and other serious metabolic disorders including type II diabetes mellitus, with increased risk for cardiovascular disease and premature death [6]–[13].

Olanzapine has high binding affinities with multiple neurotransmitter receptors including dopamine D_2_, serotonin 5-HT_2A_ and 5-HT_2C_, histamine H_1_ receptors, and muscarinic M_1_ and M_3_ receptors [8], [14]. While D_2_ and 5-HT_2A_ receptors play a critical role in the therapeutic effects of olanzapine [15], [16], evidence indicates that the H_1_, 5-HT_2C_, and M_3_ receptors are involved in antipsychotic-induced metabolic side-effects [8], [9], [17]–[24]. Strong evidence suggests that H_1_ receptor antagonism is the key factor contributing to olanzapine/clozapine-induced weight gain and obesity [9], [18], [19], [24]–[26]. In fact, a significant association of interaction between the genetic variants of H_1_ receptors (rs346074-rs346070) and BMI/obesity has been identified recently in non-affective psychotic disorder patients treated with the high-H_1_ receptor affinity antipsychotics olanzapine, clozapine and quetiapine [27].

Several animal studies have found that olanzapine could modulate histaminergic neurotransmission for the regulation of food intake and weight gain in rats [28], [29]. Further evidence showed that weight gain and obesity associated with olanzapine and clozapine are mediated by activation of the hypothalamic AMP-activated protein kinase (AMPK) pathway via blockade of H_1_ receptors [25], [30]–[32]. In fact, a recent study revealed an association between polymorphisms in the AMPK gene and weight gain induced by olanzapine and clozapine [33]. Additionally, it was reported that olanzapine down-regulates the anorexigenic neuropeptide proopiomelanocortin (POMC), but up-regulates the orexigenic neuropeptide Y (NPY), in the arcuate nuclei of the hypothalamus (Arc) [34]–[36]. Furthermore, reduced activation of the brown adipose tissue (BAT) is associated with obesity and diabetes in humans [37]. The BAT is enriched for uncoupling protein 1 (UCP_1_) [38], which is involved in olanzapine-induced weight gain observed in rat models [39]–[41]. The peroxisome proliferator-activated receptor gamma coactivator 1-alpha (PGC-1α) and PGC-1β control mitochondrial biogenesis, which plays a critical role in the BAT thermogenesis [42], and is related with olanzapine-induced weight gain [39], [41], [43]. There is evidence that activation of BAT UCP_1_ and PGC-1α are also modulated by the hypothalamic H_1_R-AMPK pathways [44], [45]. Therefore, it may be possible to control the antipsychotic-induced weight gain by modulating hypothalamic H_1_ receptors and related pathways.

Recently we found that a short-term (2 weeks) co-treatment with betahistine (an H_1_R agonist/H_3_R antagonist) and olanzapine resulted in a −45% reduction of weight gain in drug-naïve rats compared to those treated solely with olanzapine [46]. This finding was confirmed by a recent short-term (6-week) clinical trial in which first episode schizophrenia patients with a combination treatment of olanzapine, betahistine and reboxetine (a selective norepinephrine reuptake inhibitor) had significantly less weight gain than those treated with olanzapine only [47], while betahistine+reboxetine combination treatment produced a two-fold larger weight-attenuating effect than reboxetine treatment alone [47], [48].

These animal and clinical results from short-term trials supported the effects of betahistine in attenuating olanzapine-induced weight gain in drug naïve subjects [46]. It is worth noting that clinical patients suffering with schizophrenia, bipolar disease and other mental disorders often face chronic, even life-time, treatment with antipsychotic drugs [5]. Since betahistine has a very high safety profile with extremely low (1∶100,000) adverse drug reaction [49], it has a huge potential for chronic management of antipsychotic-induced weight gain and obesity in schizophrenia and other mental disorders. It is important to note that antipsychotics cause a significant body weight gain not only in drug-naïve patients, but also in chronic patients who usually have already had previous antipsychotic exposure [5], [13], [26]. However it was not clear whether chronic co-treatment of betahistine and olanzapine would have similar weight-attenuating effects, so this was addressed in this chronic animal study. Furthermore, the effects of chronic co-treatment of olanzapine and/or betahistine on the protein levels of H_1_ receptors, AMPKα, pAMPKα, NPY and POMC in the hypothalamus, as well as UCP_1_, PGC-1α and PGC-1β levels in the BAT were also investigated.

## Materials and Methods

### Animals housing and measurements

Forty-eight female Sprague–Dawley rats (201–225 g) were obtained from the Animal Resources Centre (Perth, WA, Australia). In order to reduce potential stress caused by transportation, rats were housed in pairs for 1 week prior to the start of the experiment. They were allowed *ad-libitum* access to water and standard laboratory chow diet (3.9 kcal/g; 10% fat, 74% carbohydrate and 16% protein) throughout the whole experiment. During the experiment, they were housed in individual cages under environmentally controlled conditions (22°C, light cycle from 07:00 to 19:00 and dark cycle from 19:00 to 07:00). Body weight, food intake and water intake were measured twice per week. All experimental procedures have been approved by the Animal Ethics Committee, University of Wollongong, Australia (AE11/10); and complied with the Australian Code of Practice for the Care and Use of Animals for Scientific Purposes (7^th^ edition, 2004).

### Drug preparation and treatment

Prior to drug treatment, rats were trained for oral treatment procedures by feeding cookie-dough without drugs (0.3 g) for one week. In brief, the pellets with drugs were made prior by mixing droplets of water with cookie dough powder (containing 30.9% cornstarch, 30.9% sucrose, 6.3% gelatine, 15.5% casein, 6.4% fibre, 8.4% minerals, and 1.6% vitamins) [46], [50], [51]. Controls received an equivalent pellet without drug. Rats were observed during treatment administration to ensure complete consumption of the medication pellet. Water bottles were carefully monitored for leakage, and cages were checked for uneaten food.

**Figure 1:**
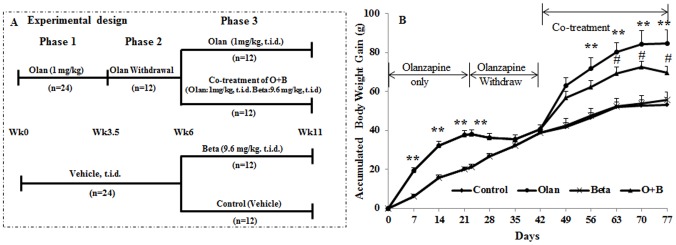
Effects of olanzapine and/or betahistine treatment on body weight gain. A: Outline of the experimental design. B: The trend of three phases of drug administration on the accumulated body weight side-effect. Olanzapine (1 mg/kg, t.i.d.; n = 12), betahistine (9.6 mg/kg, t.i.d.; n = 12), co-treatment (O+B; n = 12) or control (vehicle; n = 12) for 11 weeks. (♦: control •: olanzapine, x: betahistine, ▴: O+B co-treatment). * *p*<0.05, ** *p*<0.01 *vs.* control, # *p*<0.05 *vs.* olanzapine.

Rats were administered the treatments in 3 phases (Figure 1A). In Phase 1, 48 rats were divided into two groups during the first 3.5 weeks (Day 0–23); one half of them (n = 24) were treated with olanzapine (1 mg/kg, t.i.d.), and the other half treated with vehicle. In Phase 2, from Day 23, olanzapine was withdrawn for 19 days; all rats did not receive any treatment during this period. In Phase 3, from week 6, the two groups were divided into 4 sub-groups (n = 12) for further treatment of 5 weeks (Figure 1A): (1) olanzapine (1 mg/kg, t.i.d.), (2) co-treatment of olanzapine and betahistine, (3) betahistine (9.6 mg/kg, t.i.d.), and (4) control (vehicle). Drugs were administrated at the dosages mentioned above 3 times per day (07:00h, 14:00h, and 23:00h; with 8±1 hour interval).

After completing treatment, all rats were sacrificed (without fasting) by carbon dioxide asphyxiation. Post-mortem white adipose tissue including perirenal, periovary, inguinal and mesentery fat, sub-scapular brown adipose tissue, as well as the liver, were dissected and individually weighed [46], [52]. Body length and femur length were also measured and recorded to ascertain the effect of body growth on the body weight of rats.

### Liver histology

The liver lipid accumulation was examined using haematoxylin and eosin stains (HE; Sigma, St Louise, USA) [53], [54]. In brief, frozen livers of rats were sectioned 10 µm thick using a cryostat (LEICA, Wetzlar, Germany) and the slides were air dried at room temperature for 60 minutes. Then they were fixed with ice cold 10% formalin for 5 minutes, followed by air drying for another 60 minutes and rinsed immediately in 3 changes of distilled water. For HE staining, after drying the slides for 30 seconds at room temperature, they were placed in xylene for 1 minute, followed by 100%, 95%, 80% and 70% ethanol for 1 minute, respectively. After dipping in distilled water for 30 seconds, haematoxylin staining was performed for 5 minutes, dipping into dH_2_O again, and then placing the slides in Eosin solution for 2 minutes. The dehydration procedures were performed as follows: after the slides were dipped in dH_2_O, 70%, 80%, 95% and 100% ethanol were conducted for 30 seconds or 1 minute.

### Western blotting

Brain samples were taken 2 hours after the final drug treatment. Using the micro-dissection procedures established in our laboratory [32], [36], the hypothalamic nuclei were dissected. The dissection targeted the Arc in an overlapping pattern over the third ventricle [55]. Since the Arc is small, the punched tissue contained Arc and adjacent ventromedial nucleus (VMH); therefore the punched tissue was labelled as the mediobasal hypothalamus. The dissected brain tissue was placed into 0.5 mL Precellys Homogenising tubes and homogenised in ice-cold homogenising buffer [9.8 ml NP40 cell lysis buffer (Invitrogen, Camarillo, CA, USA), 100 µl β-Glycerophosphate (50 mM; Invitrogen), 33.3 µl PMSF (0.3M; Sigma-Aldrich, St Louis, MO, USA), and 100 µl Protease Inhibitor Cocktail (Sigma-Aldrich)]. The total protein concentrations of the tissue lysate were determined by the Bio-Rad DC Protein Assay (500-0116, Bio-Rad, Hercules, CA, USA) with bovine serum albumin (BSA) as a standard. The samples were centrifuged, and the supernatants were collected and stored at −80°C until required.

Homogenised brain samples containing 10 µg of protein were first heated at 95°C using a digital dry bath (Labnet International, USA) for 15 minutes in loading buffer containing 950 µl laemmli buffer (Bio-Rad) and 50 µl β-mercaptoethanol (Sigma-Aldrich) to denature the protein of the samples. Then, the samples were loaded into CRTGEL4–12% Bis-Tris Polyacrylamide Gels (Bio-Rad) including one channel of Precision plus Dual Colour protein Standards (Bio-Rad). The samples were subjected to electrophoresis in 1× XT-MOPS running buffer [50 ml 20× XT-MOPS running buffer (Bio-Rad) and 950 ml distilled water] at 100 V for 15 minutes followed by 200 V for 55 minutes. The separated proteins were then transferred electrophoretically onto a non-specific protein binding polyvinylidene difluoride (PVDF) membrane (Bio-Rad) in the ice cold transfer buffer (150 ml 10× Tris/Glycine Buffer (Bio-Rad), 300 ml cold methanol and 1050 ml distilled water) at 100 V for one hour. The PVDF membranes were incubated in the Tris-Buffered Saline-Tween (TBST) (Sigma-Aldrich) solution containing 5% BSA for one hour at room temperature for blocking the remaining non-specific protein binding pores on the PVDF membrane. Each membrane was then incubated in the primary antibodies including anti-AMPKα (1∶1000; Cell Signaling Technology, Beverly, MA, USA, #2532), anti-phospho-AMPKα (1∶1000; Cell Signaling, #2535) and anti-histamine H_1_ (1∶1000; Santa Cruz Biotechnology, Dallas, USA, #SC-20633), anti-POMC (1∶1000, Santa Cruz, # SC-20148), anti-UCP_1_ (1∶1000; Santa Cruz Biotechnology, #SC-6529), anti-PCG-1α (1∶1000; Santa Cruz Biotechnology, #SC-13067) and anti-PGC-1β (1∶1000; Abcam, #AB130741), which were diluted in TBST and 1% BSA buffer overnight at 4°C. Each membrane was washed 3×5 minutes in TBST buffer, followed by incubation for 1 hour at room temperature (RT) with horseradish peroxidise (HRP)-conjugated goat anti-rabbit (1∶2000; Millipore, Billerica, MA, USA) or donkey anti-goat (1∶2000, Santa Cruz Biotechnology) as secondary antibodies. The membranes were then each washed 3×5 minutes in TBST buffer at RT. The proteins of interest were visualised by reacting the membranes with Luminata Crescendo Western HRP Substrate (Millipore) via incubation, and exposing them to Amersham Hyperfilm ECL (GE Healthcare Life Science). Membranes were then re-probed with mouse anti-actin primary polyclonal antibody (1∶10000; Millipore, Temecula, CA) and HRP-conjugated rabbit anti-mouse secondary antibody (1∶3000; Millipore, Temecula, CA). The immunoreactive signals were quantified by densitometry and the values were corrected based on their corresponding actin levels. All results were normalised by taking the value of the vehicle group as 100%. Experiments were performed in duplicate.

### Enzyme immunoassay (EIA)

The NPY EIA Kit (Phoenix Pharmaceutical, USA) was performed to determine the hypothalamic NPY level using the homogenised hypothalamic Arc tissue, which was prepared for the above western blot experiments.

### Statistical analysis

Statistical analysis was performed using SPSS (version 19.0, IBM SPSS Statistics, USA). The Kolmogorov-Smirnov test was used to examine the distribution of data from all experiments. Body weight gain, food intake and water intake data from Phase 1 and 2 were analysed by two-way ANOVAs (DRUG TREATMENT×TIME as repeated measures). The Phase 3 data on body weight gain, food intake and water intake were analysed by three-way repeated ANOVAs (OLANZAPINE×BETAHISTINE×TIME as repeated measures). Two-way ANOVAs was used to compare the levels of NPY, H_1_R, AMPKα, pAMPKα, POMC, UCP_1_, PGC-1α and PGC-1β. Multiple comparisons were performed using a *post-hoc* Dunnett-T test. Pearson's or Spearman correlation tests were used to assess the relationships among these measurements. For the data without abnormal distribution, a Mann-Whitney U test was applied. All data were presented as mean ± SEM, and statistical significance was accepted when *p*<0.05.

## Results

### Effects of olanzapine and/or betahistine on weight gain, food intake and feeding efficiency

#### Phase 1. Effects of olanzapine treatment

**Figure 2:**
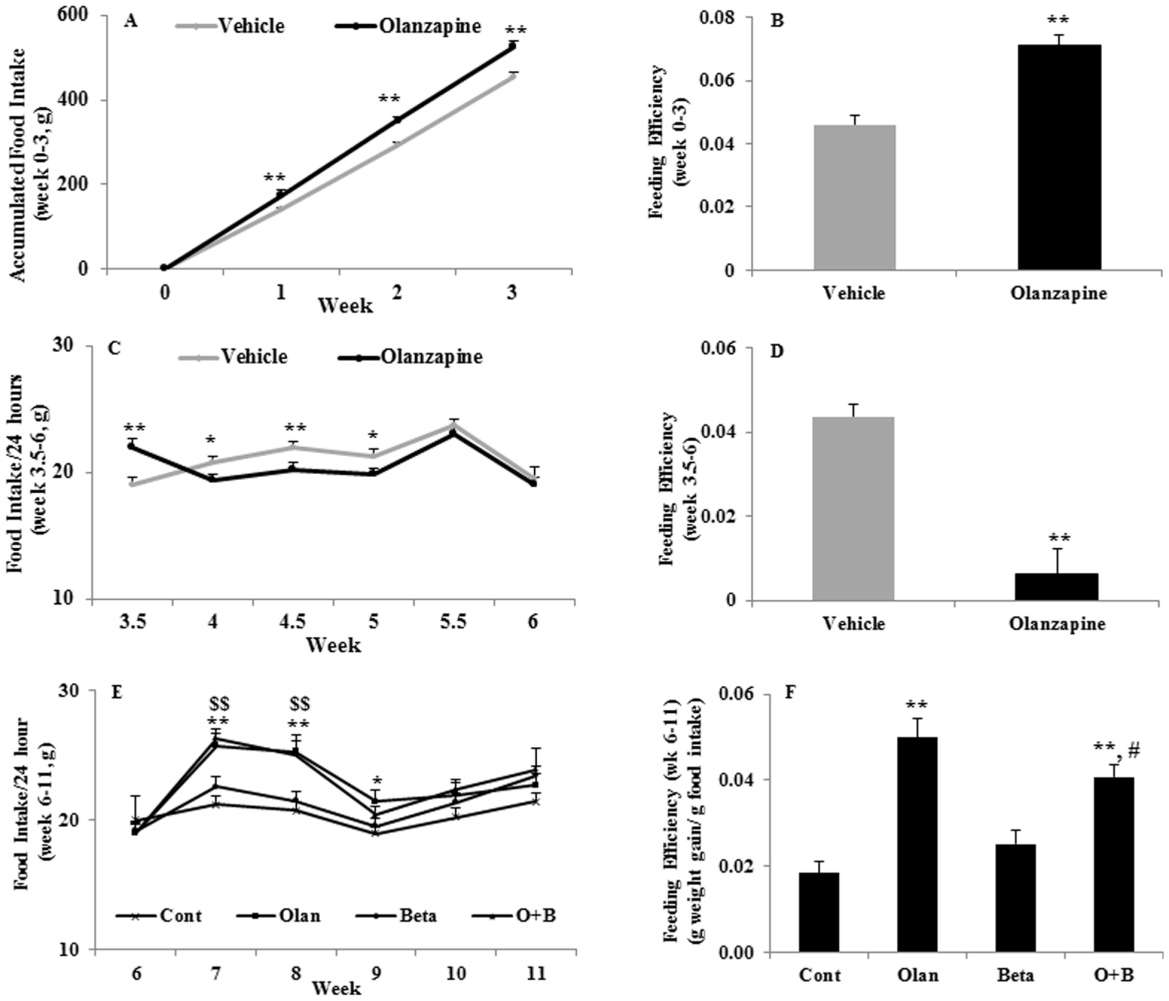
Effects of olanzapine and/or betahistine treatment on food intake and feeding efficiency. A–B: Accumulated food intake (A) and feeding efficiency (B) in the first phase of olanzapine treatment (1 mg/kg, t.i.d.; n = 12) compared with vehicles. C–D: Food intake (C) and Feeding efficiency (D) following olanzapine withdrawal. E–F: Food intake (E) and feeding efficiency (F) following chronic treatment of olanzapine (1 mg/kg, t.i.d.; n = 12), betahistine (9.6 mg/kg, t.i.d.; n = 12), co-treatment (O+B; n = 12) or vehicle (control; n = 12) for 5 weeks. (♦: control •: olanzapine, x: betahistine, ▴: O+B co-treatment). * *p*<0.05, ** *p*<0.01, olanzapine *vs.* control; $ *p*<0.05, $$ *p*<0.01, co-treatment of O+B *vs.* control; # *p*<0.05 O+B *vs.* olanzapine.

Figure 1B presents the accumulated body weight gain over the experimental period. In Phase 1, olanzapine treatment significantly increased body weight gain compared to vehicle through the treatment period of 3 weeks (all *p*<0.001) (Figure 1B). Consistent with weight gain changes, olanzapine significantly increased food intake through the treatment period (all *p*<0.05; Figure 2A). Furthermore, feeding efficiency (grams of body weight gain/grams of food intake) was significantly elevated by olanzapine treatment compared with the vehicle (*p*<0.001) (Figure 2B). However, there was no significant change of water intake in this phase (*p*>0.05).

#### Phase 2. Effect of olanzapine withdrawal

Following olanzapine withdrawal, the weight difference between the olanzapine-treated rats and vehicle were gradually narrowed: initially, olanzapine-treated rats had a significantly higher weight gain than the vehicle group (*p*<0.001), the weight loss of rats was detected following olanzapine withdrawal (Figure 1B). The weight of rats in the olanzapine group then reduced gradually to a level similar to the rats in the vehicle group after 12 days of olanzapine withdrawal (*p*>0.05), and remained at the same level as the control for the rest of the period of olanzapine withdrawal (*p*>0.05). Consistent with the changes in weight loss, olanzapine withdrawal led to a sharp decrease in food intake and remained at a lower level for 1.5 weeks compared to the vehicle group (Figure 2C), then gradually returned to a level similar to the vehicle group (Figure 2C). In contrast to olanzapine treatment, olanzapine withdrawal caused a significant decrease in feeding efficiency compared to the vehicle group (*p*<0.001) (Figure 2D). Similar to the first phase, no water intake difference between the groups was identified (*p*>0.05).

#### Phase 3. Effect of chronic betahistine co-treatment in reducing olanzapine-induced weight gain

As shown in Figure 1A, from week 7 to week 11, the rats were divided into four groups: olanzapine-only, olanzapine+betahistine (O+B) co-treatment, betahistine-only, and control (vehicle). In the olanzapine-only group, resumed olanzapine treatment significantly increased body weight gain compared to the control through the 5 weeks treatment period (all *p*<0.01; Figure 1B). On the other hand, although the O+B co-treatment group had a higher weight gain than the control and betahistine-only groups (all *p*<0.01; Figure 1B), it appeared to have a significantly lower body weight gain than the olanzapine-only group after 3 weeks' co-treatment (*p*<0.05; Figure 1B) and in total reduced −51.4% following 5 weeks' co-treatment (*p*<0.05). However, the betahistine-only treatment had no significant difference in weight gain compared to the control group (all *p*>0.05; Figure 1B). Therefore, co-treatment of betahistine and olanzapine can partly reduce/prevent weight gain induced by chronic olanzapine treatment (Figure 1B). There were no significant differences in the body or femur length among all treatment groups and the controls (Table 1), which suggested that none of the treatments affect animal growth.

**Table 1:** Mean fat mass, liver weight, and body length (mean ± SEM) in female Sprague Dawley rats treated with olanzapine (1 mg/kg, t.i.d.) and/or betahistine (9.6 mg/kg, t.i.d.) or control (vehicle).

	Control	Olanzapine	Betahistine	O+B
**Fat pad mass (g) Inguinal**	3.69±0.26	5.17±0.33**	2.85±0.20*,##	4.25±0.23#
**Perirenal**	4.95±0.45	5.54±0.40	3.75±0.37#	5.58±0.44
**Periovary**	6.81±0.54	8.82±0.65*	5.17±0.51##	8.30±0.64
**Mesentery**	2.54±0.18	3.41±0.30*	2.23±0.16##	3.03±0.20
**Brown Fat**	0.39±0.03	0.44±0.04	0.37±0.03	0.39±0.02
**Liver (g)**	12.09±0.26	13.76±0.44**	12.35±0.28##	12.78±0.39#
**Body Length (cm)**	22.30±0.21	22.54±0.13	22.71±0.15	22.41±0.10
**Femur Length (cm)**	5.26±0.04	5.19±0.02	5.24±0.03	5.20±0.02

*, *p*<0.05;

**, *p*<0.01 *vs.* control;

# , *p*<0.05;

## , *p*<0.01 *vs.* olanzapine.

The olanzapine-only treatment significantly increased food intake compared to the control for 3 weeks' of treatment (*p*<0.01; Figure 2E), then gradually reduced to a level similar to other groups. The O+B co-treatment significantly increased food intake compared with the control for the first 2 weeks' of co-treatment (*p*<0.01; Figure 2E), then it gradually declined similar to the control. Although no significant difference in food intake was detected between the O+B co-treatment group and olanzapine-only group (Figure 2E), the O+B co-treatment had a significantly lower feeding efficiency than the olanzapine-only treatment group (*p*<0.05). Therefore, O+B co-treatment was effective in decreasing feeding efficiency compared to the olanzapine-only treatment.

### Fat deposits

Compared to the control, rats with olanzapine-only treatment had a significantly higher inguinal fat mass (p<0.01), periovary fat (p<0.05), and mesentery fat (p = 0.01; Table 1). The olanzapine-only treatment group also had significantly higher inguinal fat (p<0.01), perirenal fat (p<0.05), periovary fat (p<0.01), and mesentery fat (p<0.01) than betahistine-only treatment. It is important that the rats with O+B co-treatment had significantly less inguinal fat mass than those with olanzapine-only treatment (p = 0.015) and tended to have less periovary fat (p = 0.094) and mesentery fat (p = 0.074) than olanzapine-only treatment group (Table 1). However, there was no significant difference in sub-scapula brown fat mass among all treatment groups and controls (Table 1).

### Liver weight and morphological changes

**Figure 3:**
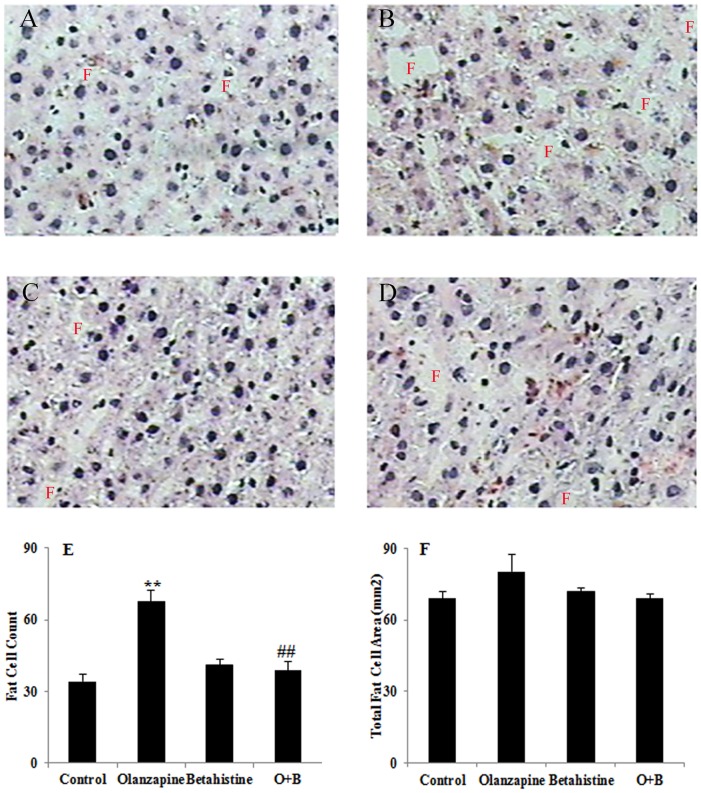
Effects of olanzapine and/or betahistine treatment (n = 12) on lipid droplet deposition of hepatic tissue. A–C: HE staining of hepatic tissue from rats treated with Vehicle (A), Olanzapine-only (B), Betahistine-only (C), and O+B co-treatment (D). E: Fat cell counts on the liver sections of different treatment groups. F: Total fat cell area measured on the liver sections of different treatment groups. ** *p*<0.01 *vs.* control; ## *p*<0.01 *vs.* olanzapine.

The rats with olanzapine-only treatment had significantly higher liver weight than controls (*p*<0.01) and those with betahistine-only treatment (*p*<0.01, Table 1). In contrast, the rats with O+B co-treatment had significantly lower liver weight than those with olanzapine-only treatment (*p*<0.05). Consistently, the HE stain showed that there was a significantly higher fat cell count in the olanzapine-only treatment group than controls, while there was a significantly lower fat cell count in the O+B co-treatment group than the olanzapine-only group (*p*<0.001; Figure 3E). In addition, the olanzapine-only group tended to have larger total fat cell areas than the control (*p* = 0.073) and the O+B co-treatment group (*p* = 0.086; Figure 3F).

### Effects of olanzapine and/or betahistine treatment on the protein expression of hypothalamic H_1_R, AMPKα, pAMPKα, NPY and POMC

**Figure 4:**
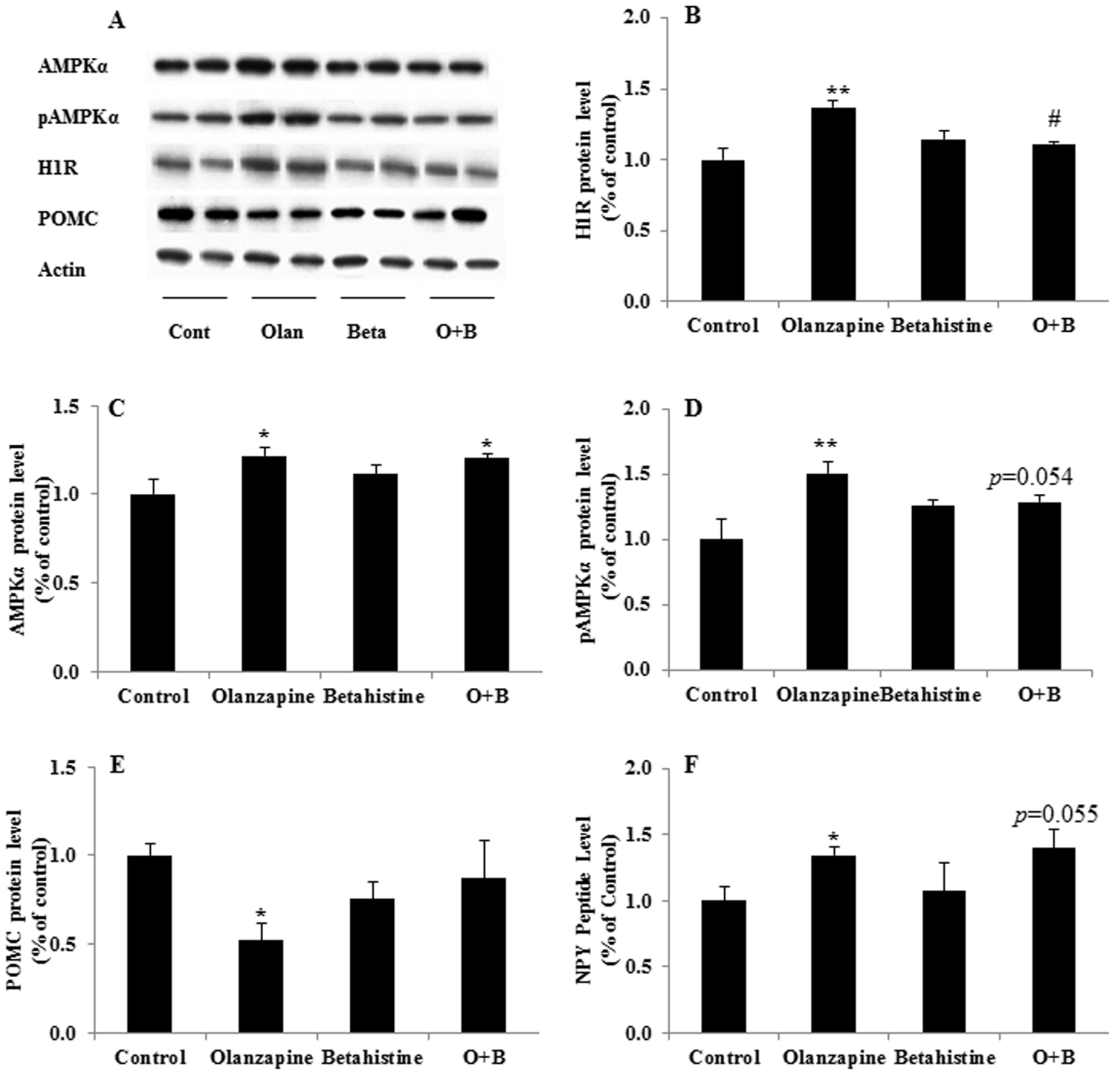
Effects of olanzapine and/or betahistine treatment on the hypothalamic protein levels of histamine H_1_R, AMPKα, pAMPKα, and POMC. A: Examples of the images of the western blot experiment showing the protein expressions of histamine H_1_R, AMPKα, pAMPKα, POMC and β-actin (n = 6). B–F: Effects of olanzapine and/or betahistine treatment on protein expressions of (B) hypothalamic H_1_R, (C) AMPKα, (D) pAMPKα, (E) POMC, (F) neuropeptide Y (NPY). Abbreviations: H_1_R: H_1_ receptor, AMPKα: AMPK-activated protein kinase α, pAMPKα: the AMPK phosphorylation α and POMC: proopiomelanocortin. * *p*<0.05, ** *p*<0.01 *vs.* control; # *p*<0.05, # *p*<0.05 *vs.* olanzapine.

Compared to the control, olanzapine treatment significantly increased the protein levels of H_1_R (+37%, *p* = 0.003; Figure 4A and B). The O+B co-treatment significantly decreased H_1_R expression compared with the olanzapine-only treatment (−26%, *p* = 0.009; Figure 4A and B). In terms of the protein expression of AMPKα, both olanzapine-only and co-treatment of O+B significantly enhanced the AMPKα level compared to the control (olanzapine only *vs.* control, +22%, *p* = 0.015; co-treatment of O+B *vs.* control, +20%, *p* = 0.025; Figure 4A and C). Both olanzapine-only treatment and co-treatment of O+B significantly enhanced the protein expression of pAMPKα compared with the control (olanzapine only *vs.* control, +51%, *p* = 0.001; co-treatment of O+B *vs.* control, +29%, *p* = 0.047; Figure 4A and D). However, the O+B co-treatment reduced the pAMPKα protein level compared with olanzapine-only treatment at a borderline significance (−22%, *p* = 0.054; Figure 4A and D). Additionally, the NPY peptide was significantly up-regulated by olanzapine-only treatment (*p* = 0.047), and co-treatment of O+B tended to elevate the NPY level compared to controls (*p* = 0.055) (Figure 4F). On the other hand, compared with the control, olanzapine-only treatment had a significant effect in decreasing hypothalamic POMC protein levels (−52%, *p* = 0.016), while co-treatment of O+B had no effect on POMC levels (*p*>0.05; Figure 4A and E).

Hypothalamic H_1_R protein expression was positively correlated with total body weight gain (*r* = 0.403, *p* = 0.028), total food intake (*r* = 0.486, *p* = 0.009) and tended to correlate with feeding efficiency (*r* = 0.207, *p* = 0.085). In addition, the hypothalamic AMPKα expression also positively correlated with body weight gain (*r* = 0.750, *p* = 0.000), total food intake (*r* = 0.553, *p* = 0.003) and feeding efficiency (*r* = 0.617, *p* = 0.001). The protein expression of hypothalamic pAMPKα was positively correlated with total body weight gain (*r* = 0.668, *p* = 0.000), total food intake (*r* = 0.515, *p* = 0.006), as well as feeding efficiency (*r* = 0.555, *p* = 0.003). There were positive correlations between hypothalamic H_1_R and AMPKα (*r* = 0.518, *p* = 0.006) and pAMPKα (*r* = 0.444, *p* = 0.017), and, there were negative correlations among hypothalamic POMC protein expression and body weight gain (*r* = −0.456, *p* = 0.014) and feeding efficiency (*r* = −0.435, *p* = 0.019). The hypothalamic NPY peptide level was positively correlated with body weight gain (*r* = 0.382, *p* = 0.036), and feeding efficiency (*r* = 0.392, *p* = 0.032).

### Effects of olanzapine and/or betahistine treatment on the protein expression of UCP_1_, PGC-1α, and PGC-1β in brown adipose tissue

**Figure 5:**
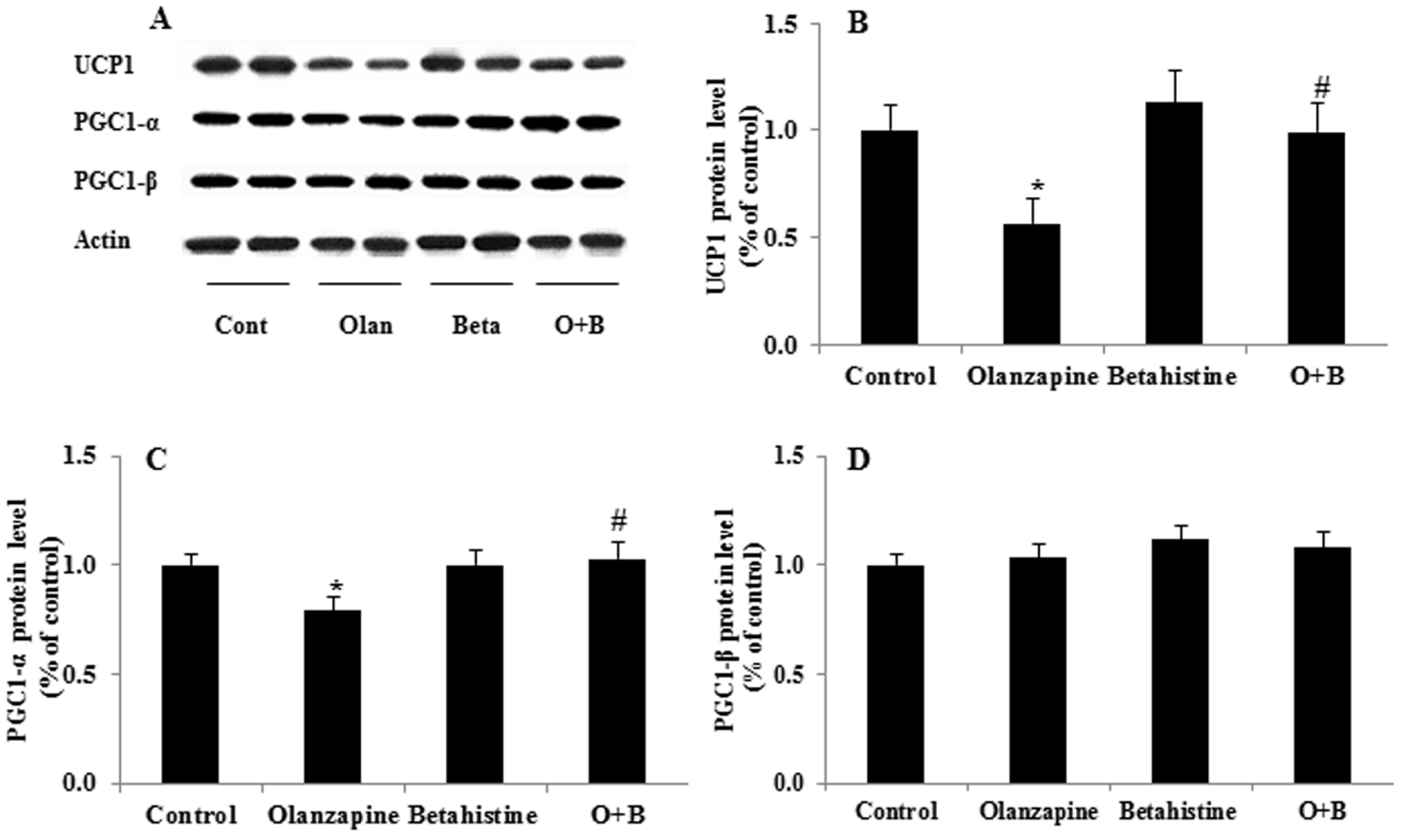
Effects of olanzapine and/or betahistine treatment on the protein levels of UCP_1_, PGC-1α, and PGC-1β in brown adipose tissue. A: Examples of the images of the western blot experiment showing the protein expressions of UCP_1_, PGC-1α, PGC-1β and β-actin (n = 6). B–D: Effects of olanzapine and/or betahistine treatment on protein expression of (B) UCP_1_, (C) PGC-1α, and (D) PGC-1β. Abbreviations: UCP_1_: uncoupling protein 1, PGC-1α: Peroxisome proliferator-activated receptor gamma coactivator 1-alpha, PGC-1β: Peroxisome proliferator-activated receptor gamma coactivator 1-beta. * *p*<0.05, ** *p*<0.01 *vs.* control; # *p*<0.05, # *p*<0.05 *vs.* olanzapine.

Olanzapine significantly down-regulated BAT UCP_1_ protein expression by 44% (*p* = 0.024), compared with the control, while co-treatment of O+B significantly reversed the decreased UCP_1_ protein level by 43% caused by the olanzapine only treatment (*p* = 0.037) (Figure 5A and B). Similarly, BAT PGC-1α protein expression was downregulated by 21% (*p* = 0.037) under olanzapine-only treatment, whilst, it was reversed significantly by co-treatment of O+B (*p* = 0.023, Figure 5A and C). However, for PGC-1β protein expression, no any significant change was observed among the treatment groups (all *p*>0.05). Additionally, BAT UCP_1_ and PGC1-α expressions in the BAT were negative correlated with hypothalamic pAMPKα levels (*r* = −0.246, *p* = 0.051; *r* = −0.374, *p* = 0.040).

## Discussion

Long term antipsychotic use remains mainstay treatment in patients with schizophrenia. Clinical trials in the past two decades have proven that, whether in first episode/antipsychotic-naïve patients or in chronic schizophrenia patients with previous antipsychotic exposure, antipsychotic administration (particularly olanzapine and clozapine) can cause significant weight gain [5], [7], [13], [56]. Similar to a previous report [57], the present study showed a withdrawal of oral olanzapine treatment also resulted in weight loss that was largely due to the decrease of food intake and feeding efficiency. Similar to the clinical findings, our results illustrated that, after drug withdrawal for over 2.5 weeks, the resumed olanzapine treatment significantly increased body weight gain [5], [15], [58], [59]. Therefore, this study provided an animal model which mimicked closely the body weight changes caused by olanzapine in drug-naïve and re-administered chronic treatment patients.

The present study was the first in a chronic animal model to detect the effect of chronic O+B co-treatment on reducing the body weight gain side-effect in subjects with chronic olanzapine exposure. The results showed that chronic O+B co-treatment produces a significant weight-attenuating effect appearing after 1 week and being statistically significant after 3-week co-treatment, with about ∼50% weight gain decrease compared to olanzapine-only treatment. Previously, a short-term study in drug-naïve rats found that 2-week O+B co-treatment significantly reduced (∼45%) body weight gain [46]. Consistently with our short-term experiment, betahistine-only treatment showed no effect on weight gain and feeding efficiency [46]. A recent clinical trial reported that antipsychotic drug-naïve schizophrenia patients with a six-week combination treatment of olanzapine (10 mg, once daily), betahistine (48 mg, t.i.d.) and reboxetine (4 mg, b.i.d.) (a selective norepinephrine reuptake inhibitor) had significantly less weight gain than those on olanzapine only [47]. In addition, a six-week trial with 3 first episode schizophrenic patients also found that betahistine (48 mg, t.i.d.) was able to prevent weight gain related to olanzapine treatment (10 mg, once daily) [60]. It is of note that both the clinical and animal studies have indicated a time-dependent effect of antipsychotic (including olanzapine)-induced weight gain. There are three stages of development of weight gain/obesity; an early acceleration stage with a rapid increase in body weight, a middle stage with continuing body weight increase following at a steadier rate, followed by a “plateau” stage maintaining a heavier weight with ongoing antipsychotic treatment [58], [61]. It is interesting that O+B co-treatment had a stronger weight gain reducing effects on the “plateau” stage (Figure 1B). Further studies are worth to investigate the effects if olanzapine dose was increased at this point, and the effects on the antipsychotics with less pronounced weight gain side-effects (as a negative control). The betahistine dosage (9.6 mg/kg rat body weight) used in this study is equivalent to ∼93 mg/kg in humans (60 kg body weight) according to dosage translation between species based on body surface area following the FDA guideline [62]. Betahistine has 3–4 hours of plasma half-life in humans with one day of urine excretion, but no data showed the half-life of betahistine in rats [63]. Although there is no data available for the half-life of betahistine in rats, it is reasonable to suppose that betahistine is most likely to have a shorter half-life in rats than in humans. Therefore, the betahistine dosage (9.6 mg/kg rat body weight) used in this study should be relevant to the human dosage (48 mg, t.i.d.) used in clinical trials [47], [60]. Taken together, results from the animal model and schizophrenia patients support the theory that both short-term and chronic co-treatment with betahistine should be effective to control olanzapine-induced weight gain in both drug-naïve subjects and those with previous antipsychotic exposure.

Consistent with the body weight changes in this study, the olanzapine-only group had more white fat mass and higher liver weight than the control and betahistine-only groups, which also corresponded with previous reports [31], [46], [51], [64], [65]. On the other hand, compared to the olanzapine-only treatment, chronic O+B co-treatment decreased inguinal fat mass and liver weight in this study. Further HE staining confirmed that olanzapine-only treatment significantly increased fat accumulation in the liver; however O+B co-treatment reduced liver fat accumulation. These results suggested that weight gain decrease in rats treated with O+B was at least partially from reduced fat accumulation. Further study is needed to investigate changes in lipid metabolism. There was no difference in body and femur length among these groups, which indicated that none of the treatments affected animal growth.

The hypothalamic nuclei, particularly the arcuate nucleus (Arc) and ventromedial hypothalamus (VMH) play crucial roles in the regulation of energy homeostasis [20], [66], [67]. Histamine H_1_R antagonists are well documented to increase appetite and obesity development [18], [68]. Several meta-analyses examined the potency of the antagonistic properties of antipsychotics for H_1_R, and the potential to utilise them to predict the likelihood of the obesity side-effect [19], [69], [70]. H_1_R antagonist properties have been identified as the main predictor for the development of antipsychotic-induced body weight gain/obesity side-effects (approximately Clozapine>Olanzapine>Risperidone>Haloperidol>Ziprasidone>Aripiprazole) [19], [20], [71], [72]. Consistent with these reports, the present study revealed that olanzapine-only treatment up-regulated the hypothalamic H_1_R levels in line with increased body weight gain and feeding efficiency/hyperphagia induced by this treatment. To our knowledge, this is the first long term animal study to investigate the effects of chronic olanzapine and betahistine co-treatment on hypothalamic H_1_R expression in the rat brain. Consistently, a recent study from our group reported that acute intracerebroventricular (ICV) injection of 2-(3-trifluoromethylphenyl histamine (FMPH; an H_1_R agonist) attenuated olanzapine induced hyperphagia [32]. It has been noted that betahistine (as a H_3_R antagonist) may increase histamine release via blocking presynaptic H_3_ autoreceptors, which could augment its direct agonistic effects on H_1_R receptors [46].

There is strong evidence that hypothalamic H_1_R and its linked AMPK signalling pathways play a crucial role in the antipsychotic-induced weight gain side-effect [18], [24], [25]. In fact, several studies have reported that olanzapine-elevated hypothalamic pAMPK was linked to its weight gain/metabolic side-effect [25], [30], [32], [73]. In this study, we found that olanzapine only increased pAMPKα and AMPKα levels in the mediobasal hypothalamus (including the Arc and VMH) compared with the control. However, the O+B co-treatment reduced pAMPKα expression compared with olanzapine-only treatment. Importantly, there were positive correlations between pAMPKα and body weight gain, food intake, feeding efficiency, as well as between AMPKα and body weight gain. Our findings were confirmed by a recent report by [31] that AMPK inhibition in the Arc reduced the olanzapine-induced weight gain side-effects in female rats by means of functional inhibition of AMPK using adenoviruses carrying dominant negative forms AMPK (DN-AMPK). This result is also in line with another study from our group that the acute ICV injection of FMPH (an H_1_R agonist) significantly attenuated olanzapine-induced AMPK levels and food intake [32]. Further investigations is needed to examine whether O+B co-treatment has different effects on AMPKα isoforms, and its downstream targets such as acetyl-CoA carboxylase (ACC) and pACC compared with olanzapine-only treatment.

The present study showed that olanzapine downregulated the protein levels of UCP_1_ and PGC-1α (biomarkers for thermogenesis), but not PGC-1β in the BAT; however these decrease were reversed by co-treatment with betahistine. The results are consistent with previous reports that the expression of BAT UCP_1_ and PGC-1α protein are decreased by chronic olanzapine treatment, which is associated with decreased energy expenditure and increased feeding efficacy/weight gain induced by chronic olanzapine treatment [39], [40]. Further studies have shown that the rapid weight gain in the early stage of antipsychotic treatment is due to a significant increase in food intake (leading to an increase in feeding efficiency), while weight gain/maintaining heavier weight following chronic treatment is largely due to decrease in energy expenditure (such as less activity and reduced thermogenesis; also leading to an increase in feeding efficiency) [39], [40], [58]. In this study, this time course was confirmed in the rats with repeated and chronic olanzapine treatment. In the chronic model, we found that chronic O+B co-treatment reduced feeding efficiency and increased BAT UCP_1_ and PGC-1α expressions (suggesting an increase of thermogenesis in BAT), but did not change food intake. Consistently, we found that chronic co-treatment with betahistine did not change the expression of hypothalamic NPY and POMC induced by olanzapine treatment. In consideration of our previous findings that the O+B co-treatment did not affect locomotor activity [46], the BAT UCP_1_ and PGC-1α changes in this study suggest that betahistine co-treatment may regulate energy expenditure by upregulating thermogenesis. Furthermore, this experiment also revealed that the BAT UCP_1_ and PGC-1α levels were negatively correlated with pAMPKα protein levels in the mediobasal hypothalamus (including the Arc and VMH). Previous studies reported that AMPK modulated BAT thermogenesis and UCP_1_ and PGC-1α expressions [36], [45], [74], [75]. As a result, it is suggested that betahistine co-treatment may regulate BAT UCP_1_ and PGC-1α through the hypothalamic H_1_R-pAMPK pathway. Therefore, these results suggest that activation of hypothalamic AMPK contributes to olanzapine-induced weight gain; however O+B co-treatment may reduce olanzapine-induced weight gain at least partly through attenuating the H_1_R-pAMPK activation, which modulates BAT UCP_1_ and PGC-1α expression and upregulates thermogenesis. Since the fasting or food intake conditions may influence the hypothalamic neuropeptides and appetite signalling pathways, the hypothalamic changes observed should considered in the context of rats sacrificed without fasting in this study.

One of limitation of this study was that plasma olanzapine levels were not monitored through the experimental periods. According to dosage translations between the species based on the body surface area following the FDA guidelines for clinical trials [51], [62], [76], the olanzapine dosage used in this project is equivalent to the recommended dosage for treating schizophrenia patients. Olanzapine has a shorter half-life in rats compared with humans. In humans, the half-life of olanzapine in plasma is 24.2 hours, compared with 72 hours in the brain [15]. However, in the rat, the half-lives of olanzapine are 2.5 hours and 5.1 hours in the plasma and brain, respectively, and the high level is retained for 8 hours after a single dose treatment trough gavage [77]. Therefore, in the present study, rats were administered with olanzapine three times/day with 8 hours intervals to ensure a consistently high concentration for better mirroring the human scenario of oral administration once per day. This treatment protocol has been proven to mimic the development of olanzapine-induced body weight in female rats [36], [46], [51], [58]. In view of the possibility that betahistine may affect olanzapine metabolism, further studies are also important to detect whether betahistine could affect plasma olanzapine levels during the O+B co-treatment period.

In this study, compared to olanzapine-only treatment group, the O+B co-treatment group showed less inguinal fat, and tended to have less periovary and mesentery fat mass, which suggests an effect of O+B co-treatment on reducing white fat mass. One technical limitation in the present study was that the white fat mass was dissected and weighed from post-mortem rat bodies. The advanced NMR (nuclear magnetic resonance) analysis may provide more detailed information about fat mass changes. Additionally, as olanzapine treatment may cause severe dyslipidemia side-effect in patients, therefore it is valuable to investigate whether O+B co-treatment could reverse olanzapine caused dyslipidemia in the future studies.

## Conclusions

To sum up, this study provides evidence in a rat model that significant body weight gain induced by olanzapine treatment could be reversed following drug withdrawal, however unfortunately weight gain resumed after re-introducing olanzapine treatment. Since patients suffering from schizophrenia and other mental disorders often require long lasting and repeated antipsychotic treatment, it is very important to control weight gain/obesity side-effects caused by chronic antipsychotic treatment. In this study, we found that co-treatment with betahistine is effective in significantly reducing weight gain induced by olanzapine through the chronic treatment course. This study further demonstrated that the mechanisms of betahistine in reducing olanzapine-induced body weight gain are through the modulation of the hypothalamic H_1_R-AMPK-BAT UCP_1_-PGC-1α pathway. Extending previous successful trials in drug-naïve subjects in both animal and first episode schizophrenia patients [46], [47], [60], this study provides further evidence to support a clinical trial to test the effectiveness of co-treatment of olanzapine and betahistine for controlling the weight gain/obesity side-effect in schizophrenia with chronic and repeated antipsychotic treatment.
